# Formation and Dissociation of CH_4_ and CO_2_ Hydrates in Presence of a Sediment Composed by Pure Quartz Mixed with Ti23 Particles

**DOI:** 10.3390/ma15041470

**Published:** 2022-02-16

**Authors:** Alberto Maria Gambelli, Giulia Stornelli, Andrea Di Schino, Federico Rossi

**Affiliations:** 1 Engineering Department, University of Perugia, Via G. Duranti 93, 06125 Perugia, Italy; federico.rossi@unipg.it; 2 Industrial Engineering Department, University of Rome “Tor Vergata”, Via del Politecnico 1, 00133 Rome, Italy; giulia.stornelli@students.uniroma2.eu

**Keywords:** gas hydrates, carbon dioxide storage, solid additives, kinetic promoters

## Abstract

The present research deals with the formation and dissociation of methane and carbon dioxide hydrates in a confined environment (small—size reactor) and in presence of a porous sediment of pure quartz impregnated with Ti23 particles. This research is part of a wider study aimed at verifying the possibility to use metallic powders, produced via gas-atomization for applications in additive manufacturing, as additives during the production/dissociation of gas hydrates. The porous medium was used to ensure the presence of Ti23 particles in the whole volume and not only in the lowest portion of the internal volume. For both the guest compounds considered, two Ti23 concentrations were explored, respectively, 8.68 and 26.04 wt%. Under the thermodynamic point of view, the dissociation process well approximated the phase equilibrium (defined with values collected from literature) for both compounds. In addition, the amount of gas trapped into hydrates, evaluated as a function of the initial amount of gas inserted inside the reactor, did not show relevant changes. Conversely, the presence of Ti23 was found to reduce the induction time for both components, thus allowing to define it as a kinetic promoter for the process. Such tendency was found to increase with the concentration.

## 1. Introduction

Gas hydrates are ice-like crystalline compounds which naturally occur in presence of natural gas molecules, which play the role of “guest” and water molecules, capable of forming a solid lattice containing the guest molecules, and commonly defined as “hosts” [1]. Since their discovery, the research on natural gas hydrates (NGH) can be distinguished in three different phases [2]. The first began with their discovery (1778–1810) and catalogued hydrates as a scientific curiosity. The second phase approximately started in 1930, where these compounds were proved to be a serious problem for the natural gas industry, due to their capability to form inside pipelines and so cause their partial or complete blockage, with consequent issues and higher costs for the natural gas transportation. Finally, in the mid-1960, hydrates began to be considered a potential new energy source [3]: numerous natural reservoirs were discovered worldwide [4], mainly in the deep oceans (97%), and for them a high energy density was theoretically proved. In particular, abundant reserves were documented in the Gulf of Mexico, South China Sea, Bearing Strait and Indian Ocean [4]. The remaining 3% of the reserves already known [5], belongs to permafrost regions; the main sites were found in Alaska, Qinghai-Tibet Plateau, Siberia and Mackenzie Delta [4]. Based on the recent estimations, the quantity of energy which can be produced with NGH is more than twice the energy still contained in all conventional energy sources currently available [6]. These estimations urged the research on this field and led to the definition of practical strategies for the natural gas recovery from hydrate reservoirs. Among them, the most diffused are depressurization, thermal stimulation and chemical inhibitor injection. To form and remain stable over time, hydrate structures need relatively high pressures and low temperatures and the specific values which these thermodynamic variables must assume, is strongly related to the guest compound it contains. The first two techniques mentioned are based on the movement of the local thermodynamic conditions, in order to cause partial dissociation of water cages, thus making the recovery of natural gas feasible. In particular, depressurization consists in lowering the local pressure, keeping the temperature constant [7,8]. The decrease of pressure will inevitably lower the temperature. Moreover, the dissociation of hydrates is an endothermic process and will produce the same effect. Lower temperatures mean higher pressure drops, to keep the process efficiency constant. These undesired effects are usually avoided by providing thermal energy from the external and thus ensuring an almost constant temperature during the process [9]. Differently from depressurization, thermal stimulation techniques consist in increasing the temperature and maintaining, at the same time, the pressure constant [10,11]. Different solutions are suitable for thermal stimulation, common examples are heat water or steam injection [12], electrical heating [13] and microwave radiation [14]. Finally, chemical inhibitors permit the shifting of the phase equilibrium curve of hydrates to higher pressures and/or lower temperatures [3]. In this way, the local thermodynamic conditions are not suitable anymore for hydrates stability and their dissociation will inevitably occur [15,16]. It has been proven that a great number of substances affect the hydrates formation and dissociation processes, both inhibiting and promoting them. The simple recovery of natural gas is almost exclusively carried out using chemical inhibitors; however, several applications may require the use of promoters [17,18], for instance the sea-water desalination via hydrate formation often needs of chemical promoter, to be economically feasible.

Chemical additives, independently from their inhibiting or promoting action, can be divided in two major groups, as a function of their area of application. The first group intervenes on the thermodynamics of the process, modifying the phase equilibrium boundary conditions of hydrates; differently, the second group alters the kinetic of the process, especially during the initial nucleation phase.

Chemical inhibitors are classified in Thermodynamic Hydrate Inhibitors (THIs) and Low Dosage Hydrate Inhibitors (LDHIs). THIs have the capability to form hydrogen bonds with water molecules, thus reducing their activity and the possibility of forming water cages around the guest compounds. Some well-known examples are methanol, glycols and salts. To be effective, high concentrations of these additives must be used; the most limiting factors for their usage are their impact on the environment and the relatively high costs. The LDHIs are distinguished in Kinetic hydrate Inhibitors (KHIs) and Anti-Agglomerants (AA). A similar classification is established for chemical promoters, which are classified in Thermodynamic Hydrate Promoters (THPs) and Kinetic Hydrate Promoters (KHIs). Similar to chemical inhibitors, these compounds are often prohibitive to the environment.

Ionic Liquids (ILs) are known for their inhibiting action on gas hydrates formation. These compounds consist of organic salts, which remain in the liquid phase at room temperature [19]. The first group of ionic liquids tested in this field, contains the imidazolium cation-based inhibitors [19]. It was established that, to be effective, ILs should be hydrophilic and their functional groups must be capable of forming hydrogen bonds with water molecules [20]. However, Pham and colleagues proved that the most accessible and available ionic liquids suitable for hydrates inhibition, are harmful [21]. In addition, their high costs cannot be considered sustainable in the oil and gas industry.

In addition, amino acids have the capability to alter the hydrate formation process. Amino acids are organic compounds and represent the building blocks of proteins [22]. They contain carboxylic acid (-COOH), amine group (-NH_2_) and a side chain, which can range from the apolar alkyl chain, which is hydrophobic, to a positive or negative charge moiety, which is hydrophilic [23].

The specific structure of the side chain strongly affects the chemical and physical properties of amino acids: for instance, it may cause strong electrostatic interaction, which creates a negative affinity between water molecules and, consequently, weakens the whole ice—such as crystalline structure. However, amino acids can also play the role of kinetic promoter during hydrates formation. In this sense, they represent a valid option in applications such as carbon dioxide (CO_2_) storage, gas mixtures separation, gas storage and transportation. In general, the specific behavior of amino acids, or their tendency to act as inhibitors or as promoters, is mainly associated to properties such as hydrophobicity, solubility in water, concentration and side chain length [24,25]. The interest on this class of additives is mainly related to their biodegradability (no environmental impact), the low costs associated to their usage and the negligible risk of corrosion for metals [26,27]. Nevertheless, their effect increases with the concentration and the overall costs are still not competitive with the classical THIs.

A further group of compounds, which shows great potentialities in hydrates promotion/inhibition, consist of nanoparticles [28]. These elements have particles whose size ranges from 1 to 100 nm [29] and are commonly classified in fullerenes, metal nanoparticles, ceramic nanoparticles and polymeric nanoparticles [30]. The nanoscale size ensures them an extremely elevated surface area and specific physicochemical properties [31,32], which make them capable of dispersing and being adsorbed at the water—gas interface, thus forming a layer which hinders the contact between hosts’ and guests’ molecules. Such property classifies the nanoparticles on the group of hydrate inhibitors; however, the presence of these particles in water enhances the heat and mass transfer rate [33,34], thus favoring the formation of hydrates [35].

Carbon nanotubes have been proven to promote hydrates formation; however, graphite nanoparticles are weak inhibitors for the process [36]. The promoting action of carbon nanotubes has been demonstrated in several researches. Park and colleagues confirmed that multi-walled nanotubes make the thermodynamic conditions required for methane (CH_4_) hydrates formation milder [37].

Zinc oxide nanoparticles were tested during carbon dioxide hydrates formation [38]. They reduced the time required for the process completion and also increased the amount of gas consumed. Conversely, the negative charge of ZnO molecules, makes these nanoparticles a slight thermodynamic inhibitor [39]. The most explored metallic nanoparticles are copper and silver. These particles are classified as promoters, because they improve the thermal conductivity of aqueous solutions. In addition to this, copper nanoparticles are also capable of lowering the induction time [40]. The same result was obtained from Pahlavanzadeh and co-workers [41]. Moreover, they established that copper nanoparticles can also improve the methane uptake and the growth rate of hydrates.

With the addition of silver nanoparticles to the aqueous solution, the induction time of methane hydrates decreased about 85%, while the quantity of gas trapped into water cages increased by 33.7% [42]. Differently, no positive effects of carbon dioxide hydrates were found with the addition of these particles [43]. More detailed information about the effect of metal oxidized nanoparticles, such as ZnO, CuO [44,45], Al_2_O_3_, TiO_2_ and Fe_2_O_3_, can be found elsewhere in the literature [46].

The chemical additive tested in this work, for the formation and dissociation of methane and carbon dioxide hydrates, can be referred to this latter groups of additives. The experiments were carried out in an aqueous solution containing Ti powder produced by gas-atomization. The size of particles places this compound outside the group of nanoparticles; however, the effect exercised on the process can be considered comparable to those produced by elements belonging to this group.

The present work is part of a wider research, focused on exploring and defining the effect of metallic particles, produced by gas-atomization and destined to applications in the additive manufacturing sector, on the formation and dissociation of gas hydrates. The use of solid particles, with diameter in the order of magnitude of micro-meters, might contribute to solve, or drastically reduce, most of the challenges attributed to chemical additives. Their physical state and their size make them recoverable, with consequent low impacts on the environment and also more contained costs, due to their possible reuse. In addition, these materials can be used to create solid reticular frameworks which can find several applications in this field. Based on this, previous experimental research were carried out and the results are briefly described here.

The same typology of experiments was previously performed using CuSn12 powder as additive for the formation/dissociation of methane and carbon dioxide hydrates [47]. Three different concentrations of this additive in the aqueous solution were tested. Independently from the type of guest compounds, these particles acted as promoters for the formation process. In presence of methane, the promoting effect during formation was noticed also at concentrations higher than 30 wt%; the same effect was also observed for lower concentrations (approximately 18 wt%) when carbon dioxide was involved in the process. In conclusion, this additive was found to be a slight promoter for methane hydrates and a good promoter for carbon dioxide hydrates, but only for concentrations above 18 wt%.

Instead, the dispersion of Inconel 718 particles in water led to different results as a function of the guest compound considered [48]. This additive promoted methane hydrates and inhibited carbon dioxide hydrates. In particular, in presence of 17.36 wt% of particles in water and at temperature of about 4 °C, the two compounds required the same pressure to form the hydrates. Finally, at concentrations higher than 26 wt% and at temperatures in the range 2.1–6.5 °C, methane hydrates occurred at milder conditions than those required for the formation of carbon dioxide hydrates. The experiments carried out with FeSi_3_ particles revealed a significant inhibiting action on carbon dioxide hydrates and a weak promotion on methane hydrates. Finally, the application of Cu particles confirmed what present in literature: this compound acted as promoter for methane hydrates. Conversely, it weakly inhibited the process in presence of carbon dioxide.

In this manuscript, the formation and dissociation of hydrates for both the types of guests as soon mentioned, were carried out and described thermodynamically and kinetically, in presence of Ti particles, dispersed in the aqueous solution. Two different concentrations were tested, respectively, 8.68 and 26.04 wt%.

## 2. Materials and Methods

### 2.1. Experimental Apparatus

**Figure 1:**
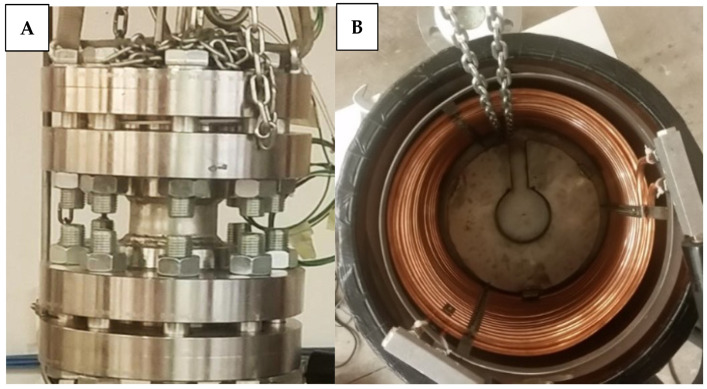
The structure of the reactor in AISI 316 stainless steel (picture (**A**)), together with the solution adopted to regulate the internal temperature (picture (**B**)), which allows to avoid relevant temperature variations, both caused from the external and by local heat productions within the same reactor.

The experimental apparatus mainly consists of a small—scale reactor (manufactured by Numanova, Terni, Italy), designed to well reproduce the formation and dissociation of gas hydrates in offshore marine reservoirs. Only the most relevant information (required for the scope of the article) are provided here, as the details (geometric and construction specifications) of this apparatus are available elsewhere in literature [49,50]. The reactor was entirely produced in AISI 316 stainless steel, in order to avoid corrosion and ensure no chemical interaction with the processes. It has cylindrical volume equal to 949 cm^3^ (height 22.1 cm and diameter 7.4 cm). The reactor is inserted in a tank (produced together with the reactor), which operates as a thermostatic bath for the system and allows to control the temperature. Being the temperature regulated from the external, the cylindrical shape ensures no temperature gradients in the radial direction. The perimetral walls of the tank are equipped with a double copper coil, where refrigerant fluid is fluxed-in from a chiller (model GC-LT, by Eurochiller, Italy). Considering the mass of the reactor (mainly concentrated in the two flanges which close the extreme sections) and the type of cooling system chosen, the apparatus is able to completely avoid interferences with the external environment and also to well counteract the internal production of heat, associated to the formation of hydrates. The following Figure 1 shows a picture of the reactor on the left and, on the right, a picture of the thermostatic bath.

The internal temperature is measured with four Type K thermocouples, produced by TC Direct and having class accuracy 1 (the declared uncertainty of these sensors is equal to ±0.1 °C), positioned at different depths inside the reactor (respectively, 2, 7, 11 and 16 cm from the edge). Considering the internal size and the quantities involved during experiments, this kind of sensor is appropriate to define an accurate temperature profile [51]. The choice of using more than one device and the specific positioning of each thermocouple, were defined by consulting what is proposed in literature [52,53]. In particular, the different depth was defined according to the following criteria: in correspondence of the first thermocouple, only the gaseous guest compound is present (before the process beginning); the second device is positioned in the water—sand mixture, slightly below the gas—liquid interface. Finally, the two other thermocouples cover the whole remaining volume: in the middle and near to the gas injection point. A digital manometer, model MAN-SD, produced by Kobold Instruments s.r.l. (with class accuracy equal to ±0.5% of full scale), was used to measure pressure and was positioned at the top. All sensors were connected to an appropriate data acquisition system and managed in LabView.

**Figure 2:**
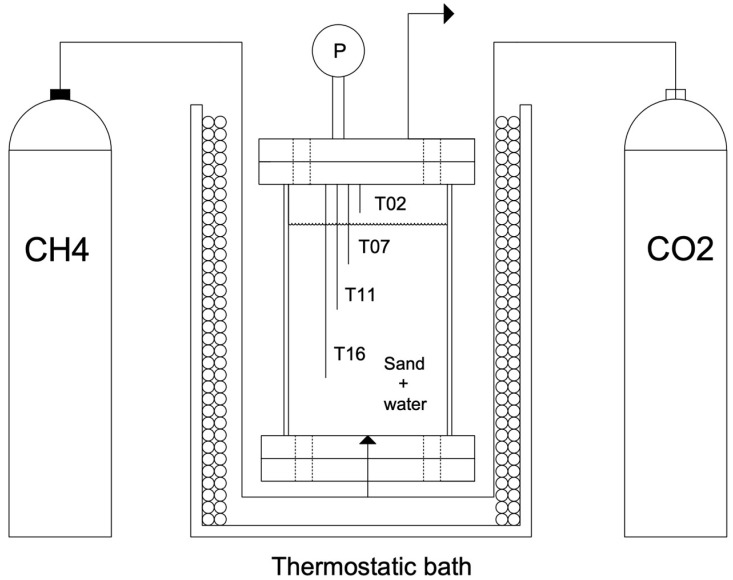
Scheme of the experimental apparatus used for the formation and dissociation of gas hydrates. Letters “P” and “T” indicate the digital manometers and the thermocouples, respectively. The four thermocouples were indicated with a specific number, which represents their depth, evaluated from the upper flange.

Figure 2 proposes a scheme of the whole experimental apparatus.

As visible in Figure 2, before entering in the reactor, the guest compound passes through a pipe immersed in the thermostatic bath. The pipe is made with copper and has high surface/volume ratio. In this way, considering the quantities required during the experiments, the gas reaches the same temperature of the reactor, before being injected in it.

### 2.2. Materials

Experiments were carried out with pure methane and carbon dioxide, having purity degree equal to 99.99%. Before gas injection, the reactor was filled with pure demineralized water and sand. The respective quantities are 236 and 744 cm^3^. Pure quartz sand was used in this work; it contains spherical size grains, having diameters in the range 90–150 µm and porosity equal to 34%. This latter parameter was evaluated with a porosimeter, model Thermo Scientific Pascal 140; it considers both the space present inside grain pores and the free space between grains. The porous medium was used to ensure a homogeneous dispersion of the Ti particles tested in this work, in the whole volume of the reactor. Without sand, these particles would have deposited on the floor. The homogeneity of this solid mixture was obtained with a mechanical stirring. The use of a transparent tank allowed to visually verify the potential heterogeneous accumulation of particles in specific sites, or its deposition on the floor of the tank. As soon none of these defects were noticed anymore, the sand—particles mixture was inserted in the reactor. The specifications of the additive are provided in Section 2.2.1.

#### 2.2.1. Ti23 Particles

**Figure 3:**
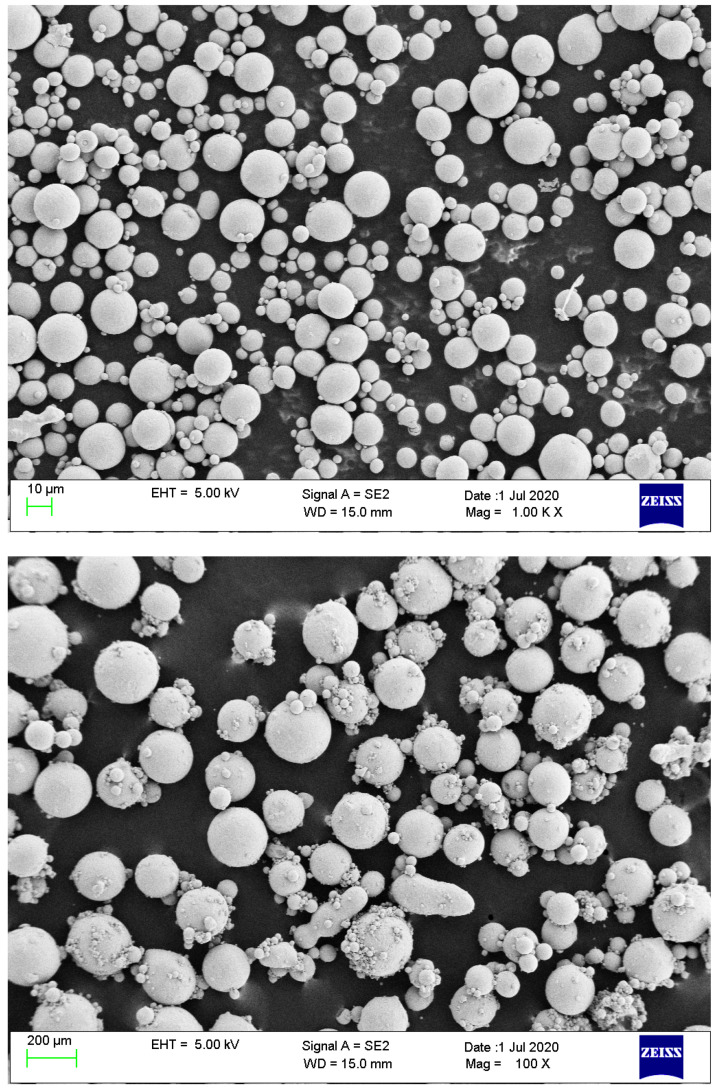

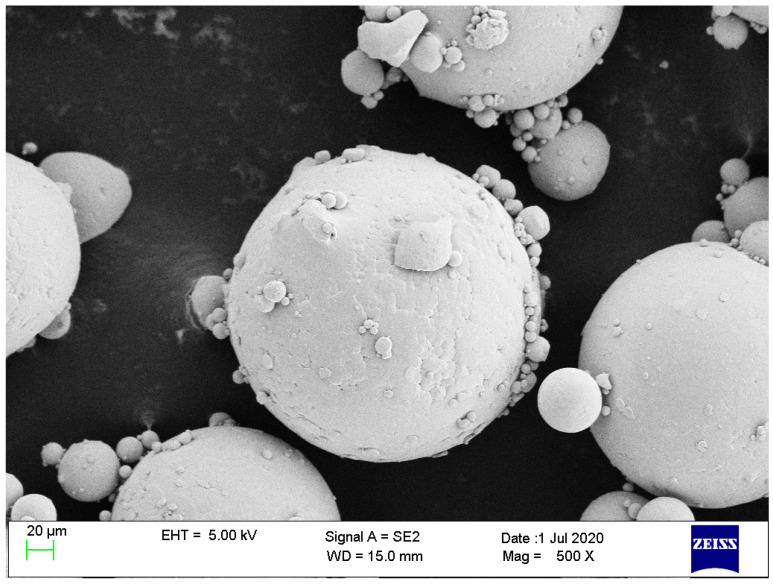
Pictures of the Ti23 powder used as additive during CO_2_ and CH_4_ hydrates formation.

Ti23 grade metallic powders are considered in this work [54]. Powders were produced by gas-atomization and have approximately spherical shape, with diameter ranging from 100 to 250 µm. These kinds of raw materials are commonly produced for applications in additive manufacturing [55,56]. The morphological characterization of particles was performed by means of high—resolution scanning electron microscope (FE-SEM Zeiss LEO-1530) and is shown in detail in Figure 3.

### 2.3. Methods

The experimental section of this work is based on eight experiments: four of them were carried out with methane as guest compound, while the remaining with carbon dioxide. For each Ti23 concentration selected, two experiments are shown. As previously asserted, the two concentrations tested in this work are 8.68 and 26.04 wt%.

**Figure 4:**
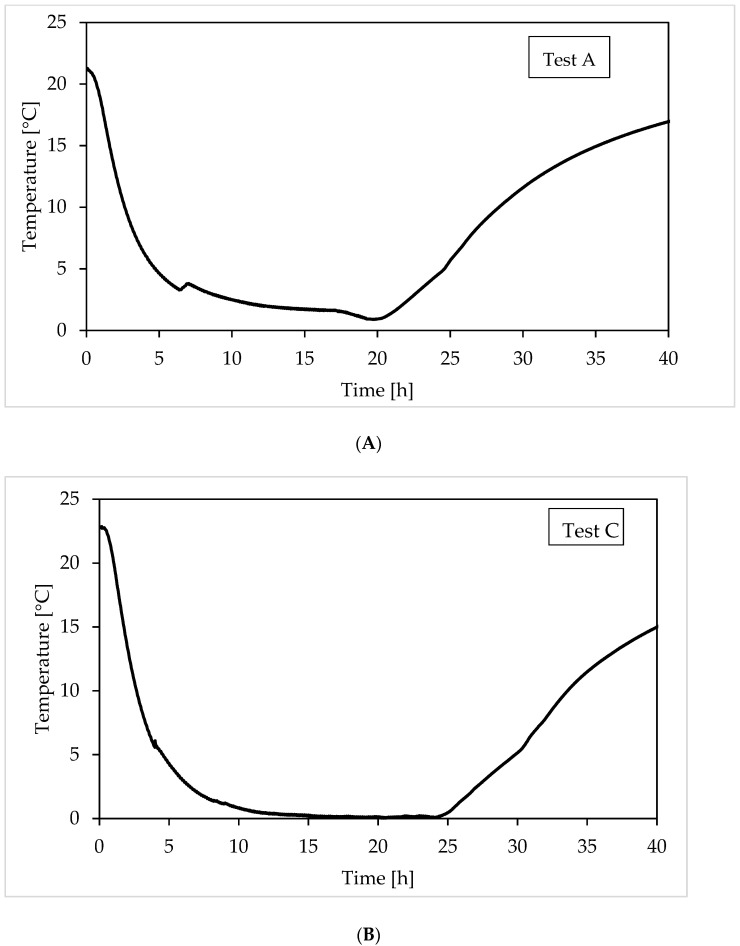
Temperature profile, controlled from the external and observed during experiments. The diagram above refers to (**A**) Test A, carried out with methane, while the diagram below describes (**B**) Test C, performed with carbon dioxide.

The methodology applied for the formation and dissociation of gas hydrates, has already been described in detail and can be easily found in literature [57,58]. The sand—Ti23 mixture was prepared outside from the reactor and then inserted inside it; water was added in a following step. Then, the reactor was closed. A spiro metallic gasket (model DN8U PN 10/40 316-FG C8 OR, provided by TecnoTubi S.r.l., Terni, Italy) was positioned between the two plates of the flange, in order to prevent possible leaks of gas. Once the reactor was closed, a weak gas stream, used as guest, was performed, in order to completely remove the air from the inlet. Finally, the ejection valve was closed and the reactor was slightly filled, until the desired pressure is reached, corresponding to 47–52 bar for methane and 41–46 bar for carbon dioxide. While pressure varied depending to the process evolution, the temperature was controlled from the external. Figure 4 shows how temperature was varied during experiments.

Every test started at temperature condition well outside the equilibrium, to ensure the absence of hydrates formation during the injection phase, which could cause uncertainties in the definition of the quantity of gas inserted inside the reactor. The chiller was regulated to gradually reach temperatures equal or slightly above 0 °C. Consequently, the temperature profile during the formation phase can be, divided in two steps: the first, during which temperature gradually decreased (as a function of the difference between its local one and the one fixed as target) and a second, where temperature remained approximately constant. During formation, some little deviation from the established trend may occur due to of the internal production of heat associated to the reaction. However, the thermal capacity of the system is enough elevated to make such deviation negligible, as shown in Figure 4. The temperature was then gradually increased, until the complete dissociation of hydrates previously formed is ensured. The pressure—temperature trend, observed during this phase, was used to thermodynamically characterize CO_2_ and CH_4_ hydrates in presence of Ti23 and to compare them with data present in the literature. These latter data were obtained from a series of experiments carried out by different researchers and using different experimental apparatuses. In particular, the values used to define the phase boundary equilibrium line for methane [59,60] and carbon dioxide hydrates [61] were taken from literature.

The process was characterized thermodynamically and kinetically. The first aspect was examined as previously explained; conversely, the kinetics of the process was shown, in the experimental section, with the pressure—temperature trend and with the evaluation of gas consumption over time.

Clearly the pressure was measured directly, while the gas consumption was calculated considering the initial quantity of gas, present inside the reactor before the beginning of hydrates production, and the amount of gas entrapped into water cages during time.

The quantities of gas were evaluated according to Equation (1):

(1) $${\mathrm{mol}}_{\mathrm{HYD}}=\frac{V_{\mathrm{PORE}}\left(P_{i}Z_{f}-P_{f}Z_{i}\right)}{Z_{f}\left(\mathrm{RT}-P_{f}/\rho_{\mathrm{HYD}}\right)}$$

In the Equation (1), V_PORE_ represents the portion of sand pores and space present between grains, available for hydrates formation, “P”, “T” and “R”, respectively, indicate pressure, temperature and the gas constant; subscripts “i” and “f” define the time period selected for the measurement. In the equation, the compressibility factor was indicated with “Z”; according to previous studies, it was calculated by using the Peng-Robinson Equation [62]. Finally, “ ρ_HYD_” represents the ideal molar density of hydrates and was defined according to the literature [63,64]. This value was defined by assuming the ideal 100% cage occupancy, which, for the process conditions verified during experiments, can be considered acceptable (see, for instance, Papadimitriou et al., (2016) [65], where the cage occupancy was provided as a function of pressure and temperature and, at the thermodynamic conditions present inside the reactor during the process, the cage occupancy well approaches 100%).

After the complete dissociation of carbon dioxide hydrates, the system did not reach the same pressure it had at the beginning of the process. This depended on the quantity of gas which remained dissolved in water. According to the literature, considering the thermodynamic conditions of a deep ocean hydrate reservoir, with time, such quantity will inevitably form hydrate and can be considered consequently [66,67].

## 3. Results and Discussion

In this section, four methane hydrates formation dissociation tests, and an equal number of experiments involving carbon dioxide, are shown and described.

**Figure 5:**
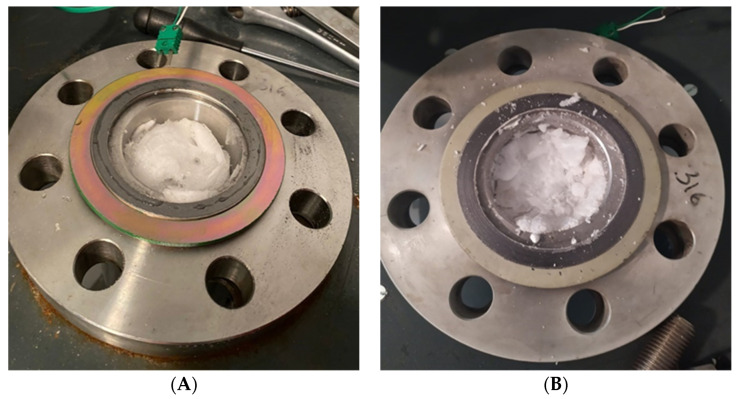
Formation of hydrates in absence (**A**) and in presence (**B**) of the porous medium. The first picture shows a less dense phase which appeared exclusively in correspondence and above the gas—liquid interface; in presence of sand, the hydrates cemented the porous medium and occupied the reactor in the region corresponding to and below the gas—liquid interface.

The presence of sand allowed to disperse the Ti23 particles along the whole length of the reactor and, at the same time, it favored the formation of hydrates in the whole reactor and not only in the interface between gas and liquid. Figure 5 reports two pictures which show the formation of hydrates in the same experimental apparatus used in this work. These give a clear idea of the function of the porous medium during experiments that will be shown in the following pages. The first, on the left, shows how hydrates form in absence of sand, while the second, on the right, is referred to hydrates’ formation in presence of sand.

The porous medium leads to the diffuse formation of hydrates, thanks to the production of numerous gas—liquid interfaces along the whole reactor. The guest compound is injected from the bottom and, before reaching the edge of the reactor, it passes through the porous sediment and remains partially trapped into its pores. It means that, if an unstirred reactor is used for experiments (as carried out in this work), a higher quantity of gas hydrates can be produced in presence of sand. Despite the overall quantity and the diffusion of hydrates inside the reactor, a relevant difference is also noticed in the morphology of hydrates. In only pure fresh water, hydrates formed at the gas—liquid interface, then they continue their growth above this interface, until occupying almost all the available volume between this interface and the top of the reactor. The mechanism is well described in [2]: hydrates form mainly at the gas—liquid interface because it ensures high concentrations of both guests and hosts molecules; moreover, the interface lowers the Gibbs free energy of nucleation.

In the following sections, methane and carbon dioxide hydrates were described thermodynamically and kinetically. The first characterization was also used to compare the results with the current literature, thus defining if the Ti23 powder promoted or inhibited the process, or none of the above.

### 3.1. Pressure and Temperature Evolution Observed during Hydrates Formation and Dissociation

**Figure 6:**
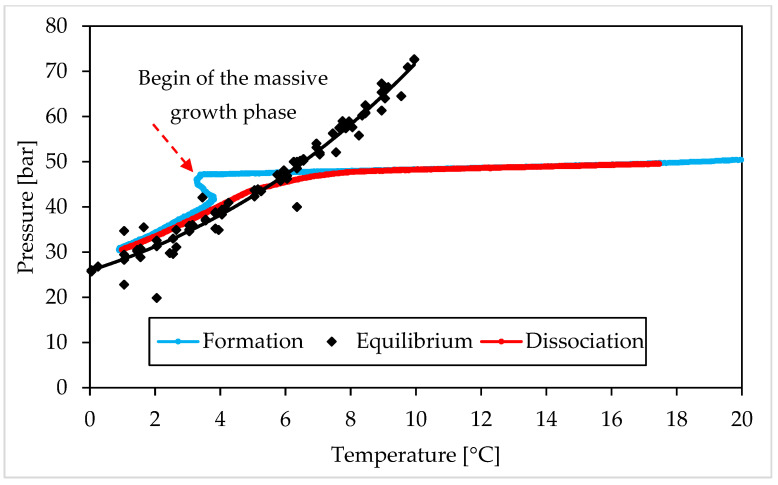
Methane hydrates formation (in blue) and dissociation (in red), observed in Test A (Ti23 equal to 8.68 wt%), compared with the phase boundary equilibrium defined in literature (in black), shown in a pressure—temperature diagram.

**Figure 7:**
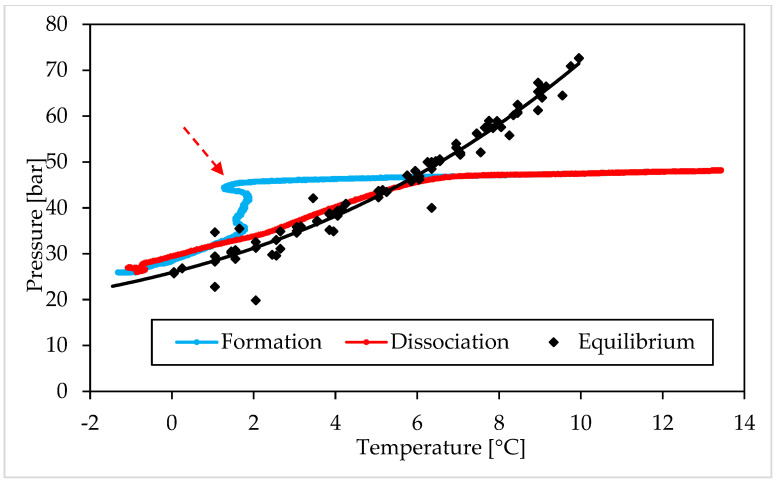
Methane hydrates formation (in blue) and dissociation (in red), observed in Test E (Ti23 equal to 26.04 wt%), compared with the phase boundary equilibrium defined in literature (in black), shown in a pressure—temperature diagram.

The following figures describe the processes of formation and dissociation of the methane hydrates. The first diagram (Figure 6) shows Test A, carried out with the lowest concentration of Ti23 selected in this study (8.68 wt%); conversely, the second diagram (Figure 7) is related to Test E, performed with the highest Ti23 concentration (26.04 wt%). The diagrams describing the remaining tests carried out with methane, are available in a Supplementary File.

This section shows and describes the P–T trend observed during experiments and compares it with equilibrium data for CO_2_ and CH_4_ hydrates. From figures, it clearly appears that experimental values, obtained during dissociation, are extremely similar to the equilibrium diagram for both the guest compounds. However, the procedure followed in this work, cannot define with exactness the real equilibrium of the system (which requires a well-defined variation of temperature and should be supported by visible confirmations produced with high-accuracy experimental apparatuses [68,69]), for two main reasons. First of all, in the case of three phase (liquid—vapor—hydrate) equilibrium, the definition of the equilibrium pressure, at a given temperature, should be made by evaluating the chemical potential of water in the liquid phase (${µ}_{W}^{L}$) and its chemical potential in the hydrate phase (${µ}_{W}^{H}$). In particular, the following condition must be verified: ${µ}_{W}^{H}$ = ${µ}_{W}^{L}$ [2]. To calculate these two quantities, parameters as Gibbs energy, molar enthalpy and molar volume for both phases, the number of cavities of a specific type for water molecule and the percentage of those cavities filled with gas, together with the activity coefficient of water in the hydrate phase, must be considered. The chemical potential for different components is usually considered to be the same in all phases; however, it is true only in conditions of equilibrium. When the number of thermodynamic variables is higher than, the number of conservation equations, the system is mathematically under determined. In such condition, the best option to establish the distribution of phases consists in defining the minimum of free energy [70]. The chemical potential of components may vary in different phases also when the system is over determined [71]. That explains why, in systems containing exclusively pure guest formers and water, the equilibrium is usually defined only vie pressure—temperature characteristics. However, the presence of a porous medium must be considered and represent a further variable to take into account. The mineral surfaces thermodynamically favour the nucleation of hydrates. In general, the presence of a porous sediment can exercise four different effects on the process [71]. When pores are excessively narrow (approximately less than 10–50 nm), the result is extra strain in the hydrate lattice. That implies higher pressures and/or lower temperatures to reach the same level of formation. Usually, the size of pores is proportional to the diameter of grains. Conversely, the presence of solid particles represents a limitation for local movements of guest molecules and makes the nucleation of hydrates more feasible and abundant [72,73]. The porous sediment also offers numerous potential sites for heterogeneous nucleation. In this sense, two opposite effects are exercised: on one hand, the surfaces of grains and their ores play the role of inhibitors, because water cannot touch them (due to the low chemical potential of water in the first adsorbed layers); on the other hand, as a function of the first effect, these surfaces act as concentrator of guest molecules and consequently represent highly suitable nucleation sites. As a conclusion, pressure and temperature are only two of several independent variables for gas hydrates in porous sediment. Thus, the equilibrium needs more information to be determined with accuracy [74]. However, the similarity of experimental results with the equilibrium conditions found in literature, give a clear idea about the extremely low degree of uncertainty produced, which does not intervene in the least on the reasonings and conclusions asserted in this work.

As visible in Figure 6 and Figure 7, each test started at thermodynamic conditions widely outside from the hydrate stability zone (or on the right of the equilibrium curve). Then, the decrease in temperature, caused from the external, moved the system to the opposite region. Here, the formation of hydrates did not occur immediately and the experimental curve continued to assume an almost linear trend, where temperature changed only as a function of the equation of state for gases. This portion of experiments allowed to define the induction time. It started when the experimental conditions entered in the hydrate stability zone and finished when the so-called catastrophic growth began; thus, it represents the phase in which the formation of hydrates did not occur, or was not enough pronounced to be detected. According to the definition, the time elapsed until the formation of a quantifiable number of hydrates, is named “induction time” [75,76]. Such parameter can be also defined as the time required to go through the metastable region [77]. The reason why during this phase gas hydrates are not capable of forming, even if the overall conditions are suitable for the process, is well explained in [2] and can be explained as a function of the excess Gibbs free energy between a solid particle of solute and the solute in solution. It consists of the sum of two parameters: the energy associated with the solute molecules which become part of the surface of the crystal and the energy related to the overall increase in volume of the crystal. The first term increases with the radius (r) of the crystal, while the second term decreases. However, the first vary with r^2^, while the second with r^3^. It means that, during the initial formation of a crystal, the Gibbs free energy tends to increase, until the crystal reaches the so-called critical size; then it gradually decreases and the catastrophic growth phase takes place.

In all tests, this latter phase can be easily distinguished in two steps: a first, during which the system tried to approach the equilibrium condition (the distance between the experimental and the equilibrium curves decreased visibly) and a second phase, where the two curves became approximately parallel and, in some cases, overlapped. During this second step, the temperature was constantly decreasing, due to the heat removal from the external. Conversely, in the first phase, as soon described, the temperature remained constant or turned back to higher values. Such trend revealed the high production rate of hydrates inside the reactor, which made the internal production of heat greater than its removal from the external. However, the formation continued and the pressure decreased, being the system widely within the hydrate stability zone (see Figure 6 and Figure 7, where the beginning of the catastrophic growth phase was highlighted with a red and dotted arrow). As visible, the beginning of catastrophic growth did not depend on the internal temperature: for instance, in Test A it started at temperature higher than 3 °C, while in Test B, carried out with the same Ti23 concentration and the same guest compound of Test A, it occurred near 0 °C.

Independently from the concentration of Ti23, the formation phase occurred similarly in all experiments. Only in Test F it seemed to be different, however the process followed the same step observed in all other tests: as soon as the induction time was finished, a visible change in slope was observed; then the process proceeded in parallel with the equilibrium curve. Finally, a more pronounced local heat production shifted the P-T curve closer to the equilibrium, until the two curves became overlapped, as in other tests.

The following dissociation phase was extremely similar in all tests: the experimental and the equilibrium diagrams were found to be overlapped in all tests and throughout the dissociation process. Only when all hydrates were dissociated, the two curves showed differences, being, since now, the experimental curve affected only by the P-T relation expressed in the equation of state for gases. The following trend made these two conclusions possible:

(i) The Ti23 powder, used as additive in this work, did not intervene on the pressure and temperature dissociation values;
(ii) The previous condition did not change with the concentration of such additive.

The following table (Table 1) shows the average of pressure values measured during experiments, the first column relates to the tests carried out in presence of 8.68 wt% Ti23, while the second column of pressure values relates to a concentration equal to 26.04 wt% Ti23.

**Figure 8:**
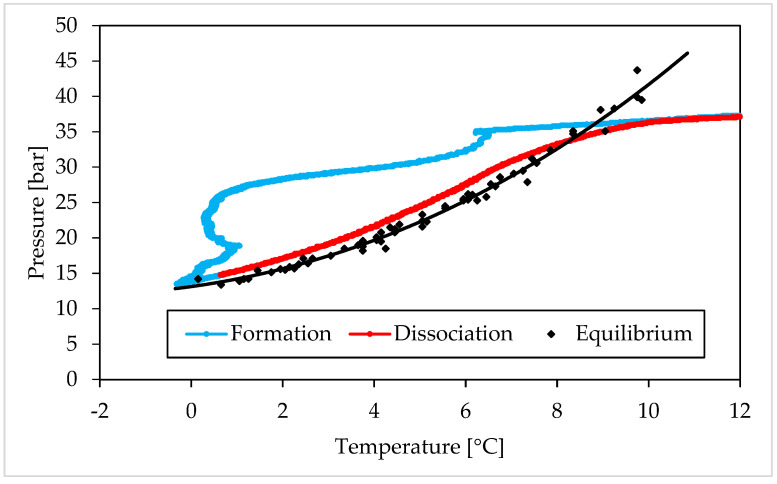
Carbon dioxide hydrates formation (in blue) and dissociation (in red), observed in Test D (Ti23 equal to 8.68 wt%), compared with the phase boundary equilibrium defined in literature (in black), shown in a pressure—temperature diagram.

**Figure 9:**
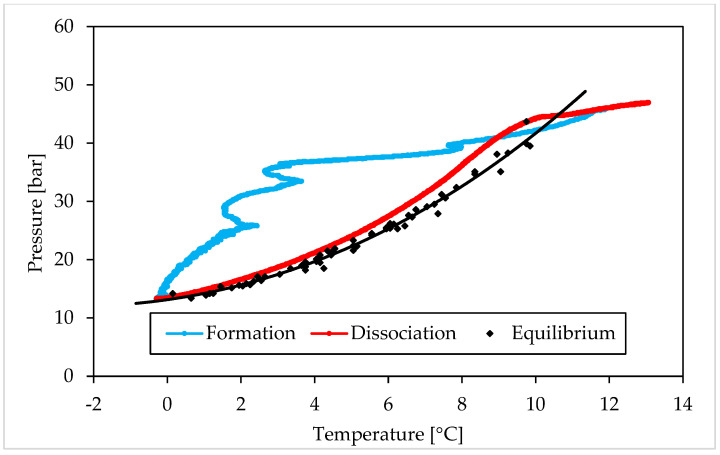
Carbon dioxide hydrates formation (in blue) and dissociation (in red), observed in Test H (Ti23 equal to 26.04 wt%), compared with the phase boundary equilibrium defined in literature (in black), shown in a pressure—temperature diagram.

Table 1 confirms that there are no relevant differences as a function of the concentration of Ti23. Figure 6, Figure 7, Figure 8 and Figure 9, together with a direct comparison of these values with data published in [3] about methane hydrates equilibrium, prove that the present additive has no effect on the P-T dissociation conditions.

The following Figure 8 and Figure 9, show the relation between pressure and temperature measured during experiments carried out with carbon dioxide.

The following diagrams revealed that, also for carbon dioxide hydrates, the same conclusions previously asserted are feasible. The massive growth occurred with a certain delay, which allowed the system to pass through the metastability zone and reach thermodynamic conditions widely within the hydrate stability zone. As expected, the formation trend was different from the one observed for methane hydrates, but it was similar in all tests involving carbon dioxide. The following dissociation phase occurred in the same way in all the experiments and well approximated the phase boundary equilibrium curve, proving that neither the presence of the additive, nor its concentration, influenced the thermodynamic of the process. Only a little deviation was observed at temperatures above 6 °C, where the experimental conditions showed slightly higher pressures than the equilibrium curve at the same temperature. However, this difference does not justify the classification of such additive as inhibitor for the system.

Additionally, for carbon dioxide hydrates, a table (Table 2) was inserted to provide the numerical values measured during experiments and used to realize the diagrams in Figure 8 and Figure 9. For each Ti23 concentration tested, the values shown in the table were defined as a mean of the results obtained in all the experiments carried out with it and, obviously, with the same guest compound.

### 3.2. Pressure, Temperature and Gas Uptake Evolution over Time

**Figure 10:**
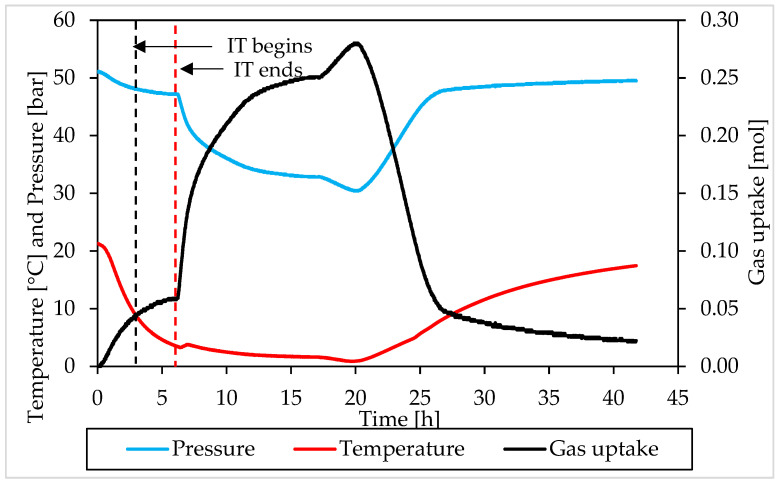
Pressure, temperature and gas uptake over time in Test A, carried out with methane and in presence of 8.68 wt% Ti23 powder.

**Figure 11:**
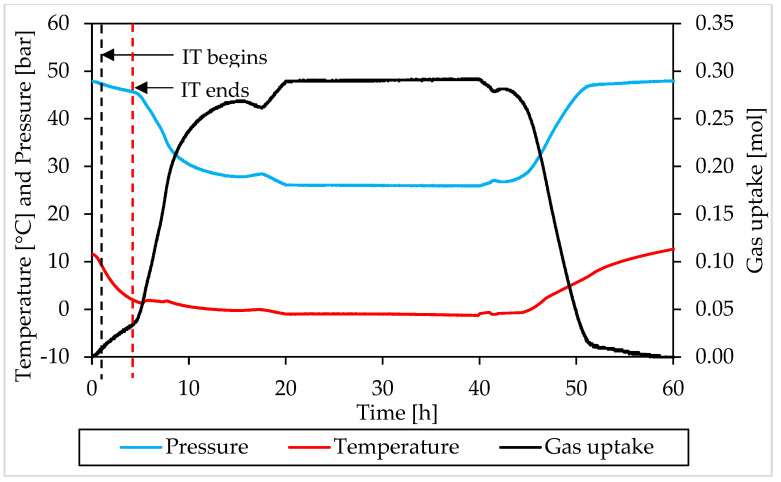
Pressure, temperature and gas uptake over time in Test E, carried out with methane and in presence of 26.04 wt% Ti23 powder.

**Figure 12:**
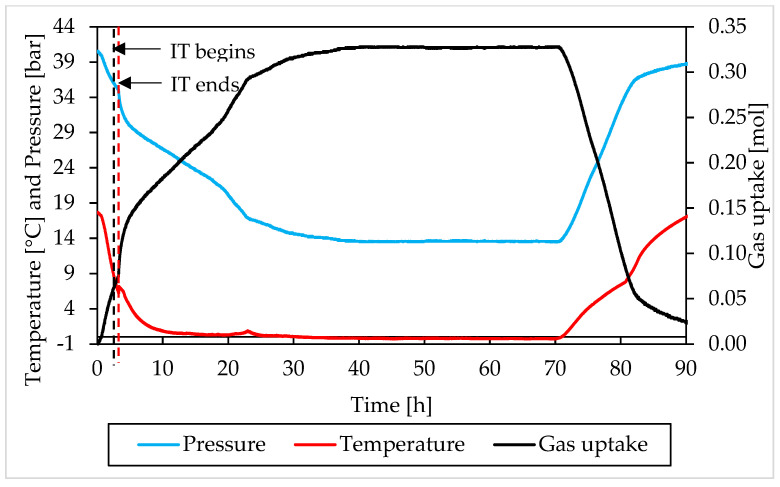
Pressure, temperature and gas uptake over time in Test D, carried out with carbon dioxide and in presence of 8.68 wt% Ti23 powder.

**Figure 13:**
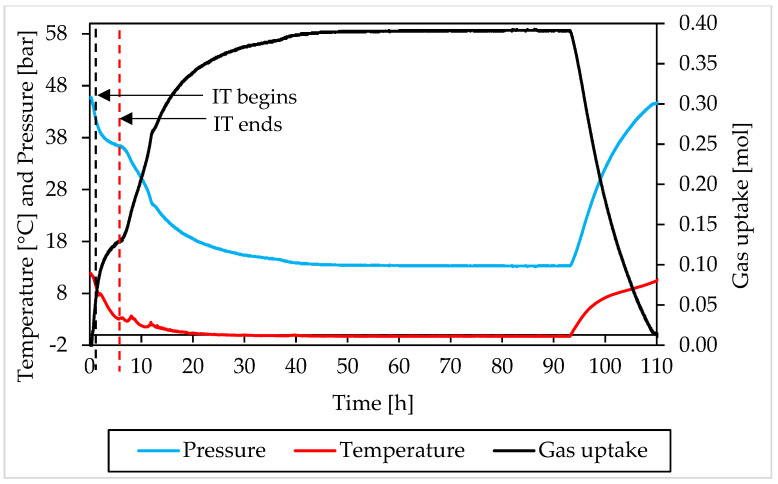
Pressure, temperature and gas uptake over time in Test H, carried out with carbon dioxide and in presence of 26.04 wt% Ti23 powder.

In this section, the formation and dissociation of hydrates, made with both components and in presence of Ti23 powder, will be described as a function of time, in order to verify if such additive affected the process or not from a kinetic point of view. In the following diagrams, the evolution of pressure, temperature and gas uptake over time was shown for each test. Figure 10 and Figure 11 describe tests involving methane, while Figure 12 and Figure 13 are associated to tests carried out with carbon dioxide.

In those diagrams, pressure and temperature were reported on the same axis: the pressure diagram was drawn in blue, while the temperature one with the red color. Finally, the gas uptake was calculated as difference between the initial amount of gas injected inside the reactor and the quantity involved into hydrates, evaluated for each measure according to Equation (1).

Furthermore, in this case, during the experiments, the temperature was controlled from the external. In all tests, a similar trend was established. The temperature started at relatively high values, to ensure no hydrate formation in this step. Then it gradually decreased, until approaching 0 °C. Then temperature remained approximately constant for a long period of time, until the process reached its completion. Finally, it was increased again and the system was shifted outside from the hydrate stability zone, in order to obtain the complete dissociation of structures previously formed.

During formation, some deviation from the fixed trend were noticed, especially at the beginning of the catastrophic growth phase of hydrates. It depends on the internal production of heat associated to the process which, in this step, became intense enough to temporarily reverse the temperature trend. This phenomenon was clearly observed in Tests A, B, C, D and H.

Moreover, during hydrates formation, the relation between pressure and temperature was not linear. For instance, when temperature remained constant, or during the last portion of this phase, the pressure firstly decreased, then it assumed the same constant trend of the temperature. The reason is obvious: in the first case, the formation of hydrates inside the reactor was still possible and consequently occurred, with the consequent entrapment of gaseous molecules; during the last phase, the process had now reached its completion. A second difference can be noticed at the incipit of the process, or before, during and, in some cases, slightly after, the induction time period: nevertheless, the fast and relevant drop in temperature, the pressure decreased slowly and accelerated its trend only at a subsequent step, when temperature had already lowered its decrease.

The trend observed for gas uptake was strongly correlated to the one of pressure, indeed this latter parameter is mostly associated to the quantity of gas molecules entrapped into the water cages. Similar results, in terms of formation, were obtained in all tests made with the same guest compound. In the tests carried out with methane, the percentage of guest compound involved in the formation of hydrates ranged from 44.2 to 59.9%; the same value was equal to 53.6–64.4% in tests made with carbon dioxide. Furthermore, for this parameter, no relevant difference was noticed in function of the concentration of Ti23 used. In most of experiments, once the dissociation phase finished, the diagram of gas uptake did not turn back to zero. In the tests made with methane, it assumed values slightly above/below zero and the difference mainly depended on the gap between the initial and the final temperature value, present inside the reactor. Differently, in the tests involving carbon dioxide, it only assumed higher values and, in some cases (for instance in Test C), the distance from zero was relevant. It depended on the capability of carbon dioxide to dissolve in water. According to what present in literature, the amount of CO_2_ dissolved in water was considered as if it was trapped into the water cages. It was previously proved that, with time, this quantity will inevitably form hydrates [66,67].

The pressure-temperature diagrams allowed to identify when the system moved outside from the stability zone and the hydrate catastrophic growth began, which coincided with the end of the induction time. The points defined in this way, were then searched and fixed in the P-t and T-t diagrams, in order to evaluate the duration of the induction time. In addition, a red and dotted vertical line was used, in Figure 10, Figure 11, Figure 12 and Figure 13, to indicate when the catastrophic growth of hydrates begun (or when the induction time finished).

Thus, the induction time was calculated as the time lapse between when the experimental curve met the equilibrium one, or when the system entered in the hydrate stability zone and when the catastrophic growth was notices in the pressure—temperature diagrams. The results obtained are shown in Table 3.

The uncertainty on the evaluation of the induction time is mainly associated to the sampling rate, which is equal to one measure every thirty seconds; thus, it can be considered equal to ±0.01 h.

The induction time is defined as the time lapse necessary to make the formation of hydrates enough intense to be detected; however, from diagrams, it seems that such formation was visible also before and during that time lapse. When defining this parameter, the pressure drop associated with the decrease in temperature should also be considered and, approximately, evaluable with the equation of state for gas. Thus, both during formation and dissociation phases, the variation in pressure is due to the sum of two different contributions: the formation/dissociation of hydrates and the variation of pressure with temperature. As clearly visible in the P-T diagrams (Figure 6, Figure 7, Figure 8 and Figure 9), during the induction time, the internal pressure decreases; however, this pressure drop was exclusively due to the second contribution. As soon as the catastrophic growth occurred, the pressure started to decrease faster and the pendency of its diagram changed consequently.

The changes in the induction time period represent the most relevant effect exercised on the process by Ti23 particles: this parameter was found to decrease with the concentration of Ti23 in the system. For methane hydrates, the induction time was equal to 2.75–6.66 h in presence of 8.68 wt% Ti23, while it was in the range 1.38–2.93 h in presence of 26.04 wt% Ti23. Similarly, for carbon dioxide hydrates, the induction time passed from 0.82–0.9 h (8.68 wt%) to 0.3–0.61 h (26.04 wt%). While the absolute value measured during tests may be affected by several variables and change consequently (see, for instance, the difference observed between Test A and Test B, carried out with the same guest and with the same concentration of Ti23), its decreasing trend, depending on the additive concentration, is clear and well visible from experimental results.

As a conclusion of the present research, it was found that the insertion of solid Ti23 particles on the hydrates’ formation environment, does not produce any significant variation in the thermodynamic of the process, neither for methane nor for carbon dioxide hydrates. The pressure and temperature values, measured during hydrates dissociation, are strongly agreed with the phase equilibrium values present in literature. Such condition remained unchanged with the concentration of Ti23. Differently, this additive intervened on the kinetic of the process. In particular, it was capable of reducing the induction time of the process. The initial difference between methane and carbon dioxide was already known and proved [78], however, for both compounds, the additive produce a relevant reduction of the induction time, as shown in Table 3.

While this research has mainly focused on the effects of this additive on the process, further works will be focused on accurately defining of the properties of Ti23 which made it possible (thermal conductivity, surface roughness and so on). Moreover, further experiments, carried out with different Ti23 concentrations, will be produced to define, with accuracy, the relation between the induction time and the additive concentration on the formation environment.

## 4. Conclusions

The present research deals with the formation and dissociation of methane and carbon dioxide hydrates in pure demineralized water and in presence of a silica-based porous sediment, impregnated with Ti23 solid particles. Topic of the research was to define the effect of this additive (Ti23) on the process.

Several tests were performed with both compounds and two different Ti23 concentrations were tested, 8.68 wt% and 26.04 wt%. The processes were firstly described thermodynamically; afterwards, the kinetic of the process was taken into account. In particular, the induction time was evaluated and the pressure, temperature and gas uptake behavior as a function of time, were analyzed. The following conclusions were possible:

(i) The addition of Ti23 particles to the system did not cause relevant changes in the thermodynamic of the process: in all tests and independently from the gaseous guest considered, the dissociation curve approached the phase equilibrium diagram during the whole process;
(ii) In addition, during hydrates formation, none difference in the thermodynamic behavior, as a function of the quantity of Ti23 used, was noticed;
(iii) Similarly, the analyses carried out in this work, did not highlight any relation between the presence of Ti23 (and its concentration) and the quantity of hydrates formed;
(iv) The induction time was found to be different, using the same apparatus, between methane and carbon dioxide hydrates. However, the presence of Ti23 was found to significantly decrease this time period. Moreover, results suggested that this effect is proportional to the concentration of Ti23 in the system. The accelerating effect exercised by Ti23 on the initial portion of hydrates formation appeared clearly and was confirmed in all experiments. In conclusion, the results, produced and described in this work, led us to define that Ti23 powder is a neutral additive for the thermodynamics of methane and carbon dioxide hydrates. Furthermore, it is capable of reducing the time lapse from when the system enters in the hydrate stability zone to when the massive growth phase occurs.

## Figures and Tables

**Table 1:** Dissociation values obtained for methane hydrates at different Ti23 concentrations.

	Ti23 8.68 [wt%]	Ti23 26.04 [wt%]		Ti23 8.68 [wt%]	Ti23 26.04 [wt%]
Temperature [°C]	Pressure [bar]	Pressure [bar]	Temperature [°C]	Pressure [bar]	Pressure [bar]
0.0	-	29.08	3.5	38.46	38.64
0.1	-	29.49	3.6	38.86	38.96
0.2	-	29.83	3.7	39.26	39.3
0.3	-	29.98	3.8	39.52	39.6
0.4	-	30.18	3.9	39.93	40
0.5	-	30.42	4.0	40.32	40.25
0.6	-	30.6	4.1	40.6	40.51
0.7	-	30.93	4.2	41	40.92
0.8	-	31.21	4.3	41.33	41.26
0.9	-	31.43	4.4	41.74	41.49
1.0	30.59	31.63	4.5	42.08	41.82
1.1	30.92	32.02	4.6	42.41	42.08
1.2	31.2	32.18	4.7	42.8	42.58
1.3	31.43	32.43	4.8	43.07	42.99
1.4	31.62	32.7	4.9	43.49	43.21
1.5	31.99	32.91	5.0	43.76	43.49
1.6	32.18	33.08	5.1	43.89	43.81
1.7	32.59	33.32	5.2	44.08	44
1.8	32.83	33.48	5.3	44.3	44.3
1.9	33.18	33.67	5.4	44.41	44.49
2.0	33.39	33.89	5.5	44.57	44.7
2.1	33.8	34	5.6	44.81	44.98
2.2	34.08	39.17	5.7	44.9	45.22
2.3	34.41	34.58	5.8	45.09	45.38
2.4	34.83	34.91	5.9	45.3	45.57
2.5	34.99	35.17	6.0	45.49	45.78
2.6	35.39	35.39	6.1	45.57	45.97
2.7	35.81	35.8	6.2	45.79	46.19
2.8	36.08	36.17	6.3	45.98	46.28
2.9	36.5	36.58	6.4	46.06	46.39
3.0	36.83	36.9	6.5	46.28	46.55
3.1	37.09	37.31	6.6	46.39	46.69
3.2	37.5	37.51	6.7	46.56	46.8
3.3	37.9	37.98	6.8	--	46.83
3.4	38.17	38.3	6.9	--	46.88

**Table 2:** Dissociation values obtained for carbon dioxide hydrates at different Ti23 concentrations.

	Ti23 8.68 [wt%]	Ti23 26.04 [wt%]		Ti23 8.68 [wt%]	Ti23 26.04 [wt%]
Temperature [°C]	Pressure [bar]	Pressure [bar]	Temperature [°C]	Pressure [bar]	Pressure [bar]
0.0	-	13.9	5.0	23.9	24.31
0.1	-	13.98	5.1	24.39	24.48
0.2	-	14.09	5.2	24.59	24.79
0.3	-	14.17	5.3	24.79	25.15
0.4	-	14.3	5.4	25.06	25.48
0.5	-	14.38	5.5	25.28	25.89
0.6	-	14.57	5.6	25.4	26.08
0.7	-	14.65	5.7	25.56	26.46
0.8	-	14.89	5.8	25.67	26.79
0.9	-	14.97	5.9	25.89	27.07
1.0	15.86	15.16	6.0	25.97	27.57
1.1	15.97	15.3	6.1	26.08	27.9
1.2	15.99	15.46	6.2	26.3	28.31
1.3	16.13	15.57	6.3	26.35	28.5
1.4	16.17	15.78	6.4	26.42	29
1.5	16.35	15.86	6.5	26.57	29.42
1.6	16.55	16.05	6.6	26.65	29.91
1.7	16.77	16.27	6.7	26.79	30.27
1.8	16.88	16.46	6.8	27.19	30.6
1.9	17.04	16.63	6.9	27.83	31.09
2.0	17.15	16.88	7.0	28.26	31.52
2.1	17.37	16.96	7.1	28.81	31.91
2.2	17.46	17.29	7.2	29.33	32.32
2.3	17.64	17.37	7.3	29.7	32.7
2.4	17.78	17.64	7.4	30.19	33.19
2.5	18.05	17.85	7.5	30.62	33.67
2.6	18.27	18.04	7.6	31.07	34.09
2.7	18.46	18.13	7.7	31.41	34.59
2.8	18.65	18.46	7.8	31.83	35.11
2.9	18.87	18.65	7.9	32.09	35.51
3.0	19.06	18.87	8.0	32.57	36.09
3.1	19.35	19.14	8.1	32.86	36.58
3.2	19.55	19.35	8.2	33.16	37.1
3.3	19.77	19.63	8.3	33.4	37.66
3.4	19.97	19.86	8.4	33.71	38.31
3.5	20.28	20.05	8.5	34.17	38.66
3.6	20.47	20.28	8.6	34.46	39.37
3.7	20.81	20.56	8.7	34.58	39.74
3.8	20.89	20.89	8.8	34.7	40.41
3.9	21.16	21.09	8.9	-	40.82
4.0	21.49	21.38	9.0	-	41.34
4.1	21.66	21.58	9.1	-	41.74
4.2	21.99	21.91	9.2	-	42.09
4.3	22.18	22.18	9.3	-	42.42
4.4	22.48	22.41	9.4	-	42.81
4.5	22.68	22.82	9.5	-	43.08
4.6	22.89	22.97	9.6	-	43.41
4.7	23.17	23.3	9.7	-	43.6
4.8	23.49	23.66	9.8	-	43.72
4.9	23.66	23.91	9.9	-	43.83

**Table 3:** Duration of the induction time for each test.

Test n°	Guest	Ti23 [wt%]	Induction Time [h]
A	CH_4_	8.68	2.75
B	CH_4_	8.68	6.66
C	CO_2_	8.68	0.82
D	CO_2_	8.68	0.9
E	CH_4_	26.04	2.93
F	CH_4_	26.04	1.38
G	CO_2_	26.04	0.61
H	CO_2_	26.04	0.3

## Data Availability

Not applicable.

## Supplementary Materials

The following supporting information can be downloaded at: https://www.mdpi.com/article/10.3390/ma15041470/s1, Figure S1: Methane hydrates formation (in blue) and dissociation (in red), observed in Test B (Ti23 equal to 8.68 wt%), compared with the phase boundary equilibrium defined in literature (in black), shown in a pressure–temperature diagram; Figure S2: Methane hydrates formation (in blue) and dissociation (in red), observed in Test F (Ti23 equal to 26.04 wt%), compared with the phase boundary equilibrium defined in literature (in black), shown in a pressure–temperature diagram; Figure S3: Carbon dioxide hydrates formation (in blue) and dissociation (in red), observed in Test C (Ti23 equal to 8.68 wt%), compared with the phase boundary equilibrium defined in literature (in black), shown in a pressure–temperature diagram; Figure S4: Carbon dioxide hydrates formation (in blue) and dissociation (in red), observed in Test G (Ti23 equal to 26.04 wt%), compared with the phase boundary equilibrium defined in literature (in black), shown in a pressure–temperature diagram; Figure S5: Pressure, temperature and gas consumption over time in Test B, carried out with methane and in presence of 8.68 wt% Ti23 powder; Figure S6: Pressure, temperature and gas consumption over time in Test F, carried out with methane and in presence of 26.04 wt% Ti23 powder; Figure S7: Pressure, temperature and gas consumption over time in Test C, carried out with carbon dioxide and in presence of 8.68 wt% Ti23 powder; Figure S8: Pressure, temperature and gas consumption over time in Test G, carried out with carbon dioxide and in presence of 26.04 wt% Ti23 powder.
