# Supplementary material for: Formation and Dissociation of CH4 and CO2 Hydrates in Presence of a Sediment Composed by Pure Quartz Mixed with Ti23 Particles
Source: Materials (Basel). 2022 Feb 16;15(4):1470. doi: 10.3390/ma15041470 (PMC8877296; doi:10.3390/ma15041470)
Supplement: Supplementary file 1 [file materials-15-01470-s001.zip › materials-1574119-supplementary.pdf]

materials

Supplementary File

Formation and Dissociation of CH_4 and CO_2 Hydrates in Presence of a Sediment Composed by Pure Quartz Mixed with Ti23 Particles

Alberto Maria Gambelli [1,*], Giulia Stornelli [2], Andrea Di Schino [1,*] and Federico Rossi [1]

Pressure – Temperature diagrams of Test B and Test F.

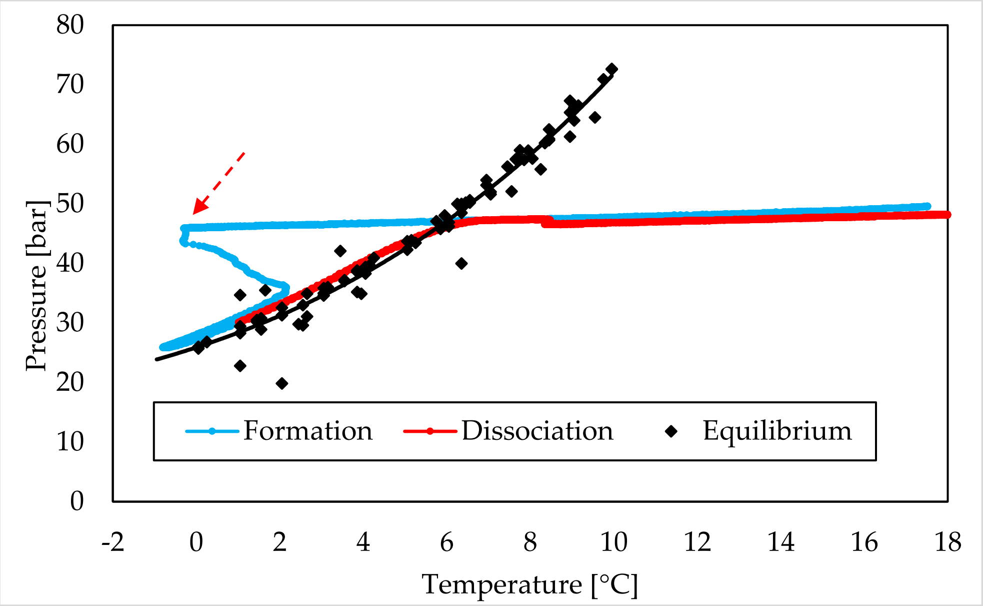

Figure S1. Methane hydrates formation (in blue) and dissociation (in red), observed in Test B (Ti23 equal to 8.68 wt%), compared with the phase boundary equilibrium defined in literature (in black), shown in a pressure – temperature diagram.

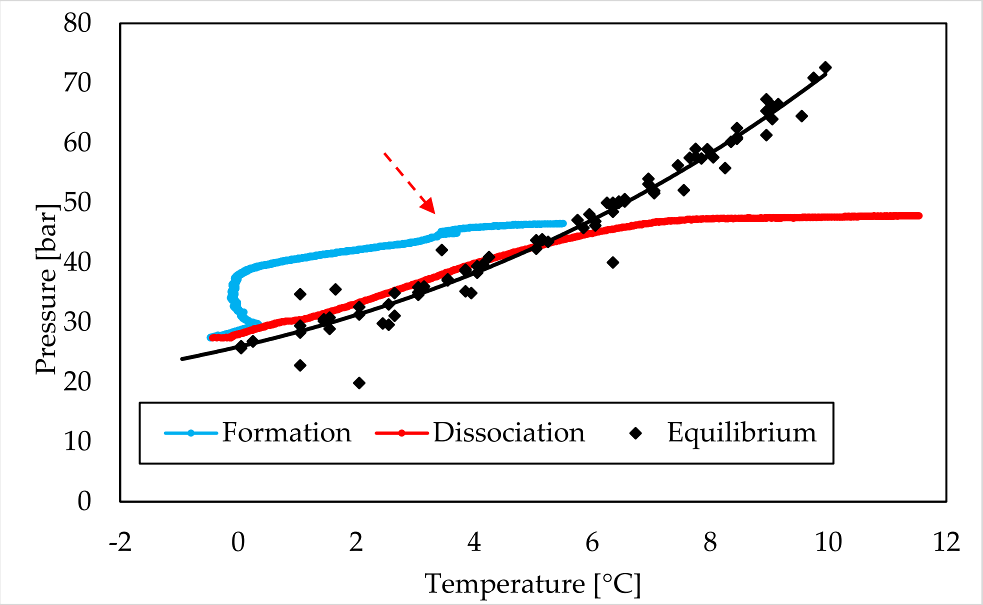

Figure S2. Methane hydrates formation (in blue) and dissociation (in red), observed in Test F (Ti23 equal to 26.04 wt%), compared with the phase boundary equilibrium defined in literature (in black), shown in a pressure – temperature diagram.

Pressure – Temperature diagrams of Test C and Test G.

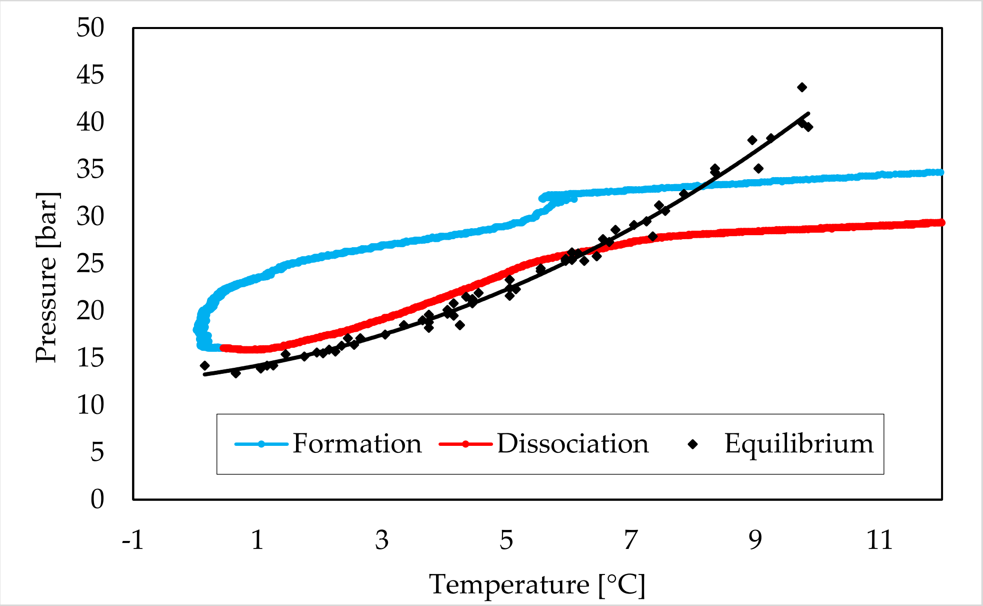

Figure S3. Carbon dioxide hydrates formation (in blue) and dissociation (in red), observed in Test C (Ti23 equal to 8.68 wt%), compared with the phase boundary equilibrium defined in literature (in black), shown in a pressure – temperature diagram.

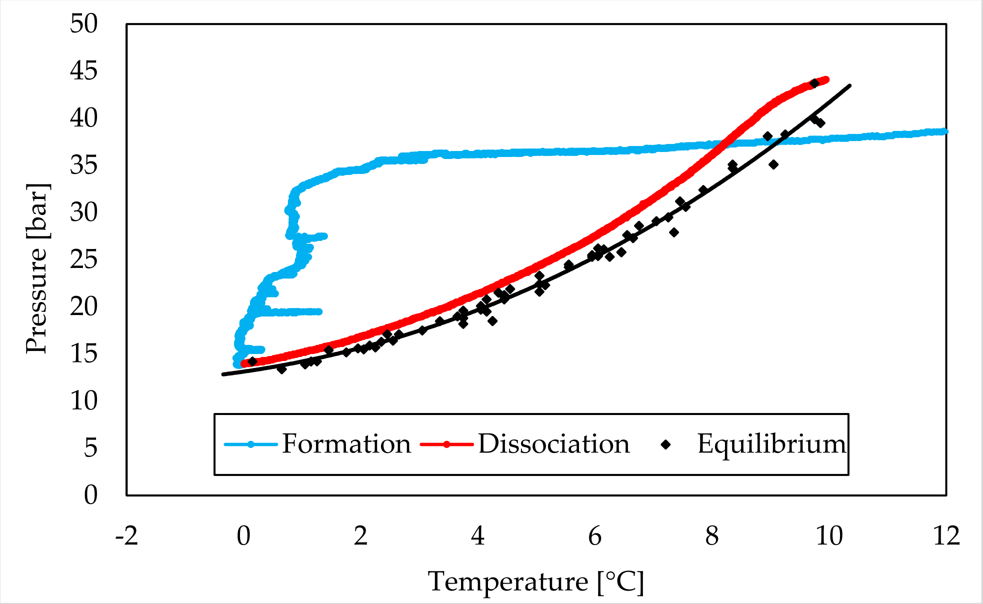

Figure S4. Carbon dioxide hydrates formation (in blue) and dissociation (in red), observed in Test G (Ti23 equal to 26.04 wt%), compared with the phase boundary equilibrium defined in literature (in black), shown in a pressure – temperature diagram.

Pressure, temperature and gas consumption over time of Test B and Test F.

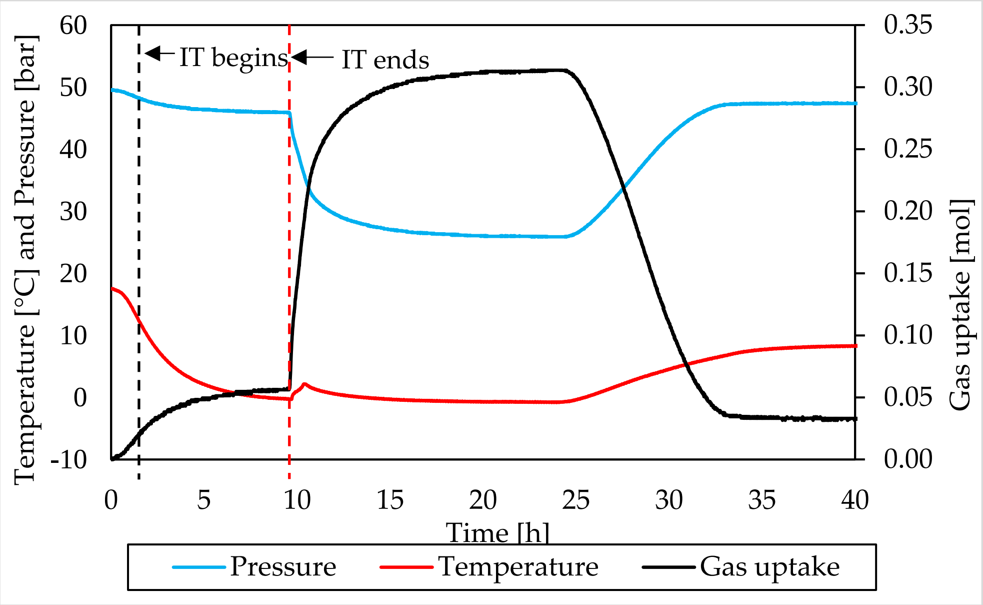

Figure S5. Pressure, temperature and gas consumption over time in Test B, carried out with methane and in presence of 8.68 wt% Ti23 powder.

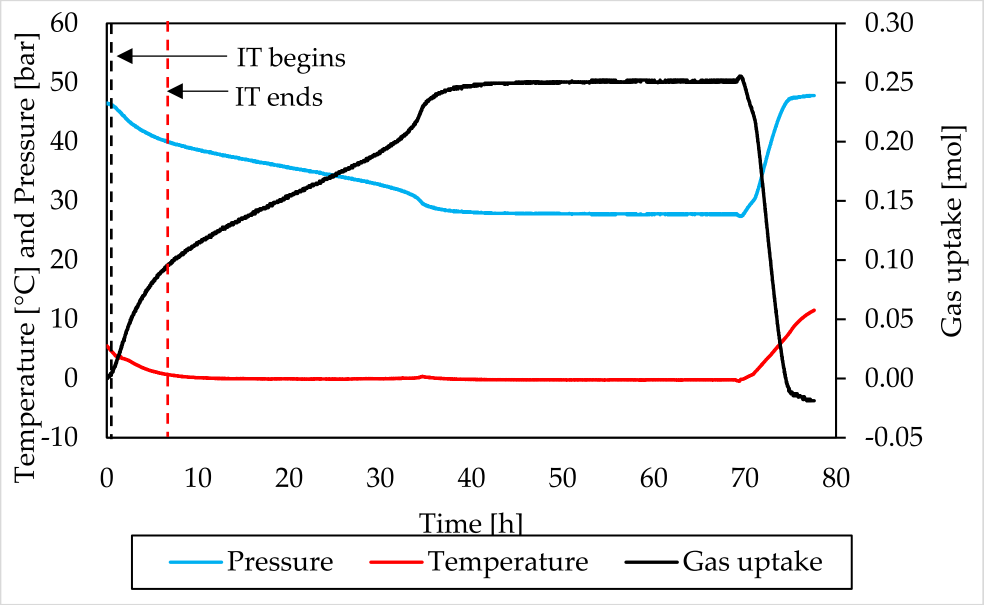

Figure S6. Pressure, temperature and gas consumption over time in Test F, carried out with methane and in presence of 26.04 wt% Ti23 powder.

Pressure, temperature and gas consumption over time of Test C and Test G.

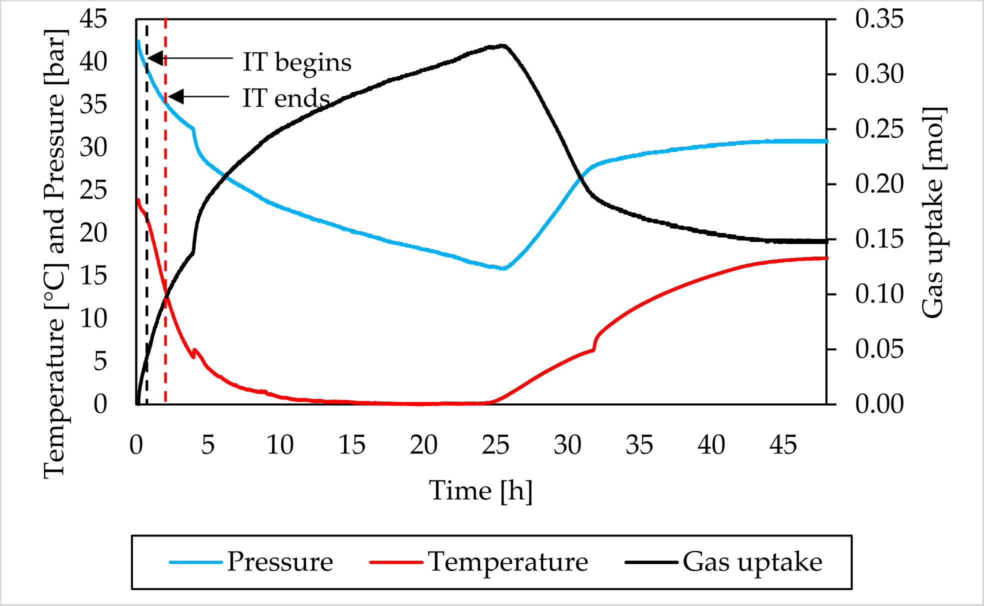

Figure S7. Pressure, temperature and gas consumption over time in Test C, carried out with carbon dioxide and in presence of 8.68 wt% Ti23 powder.

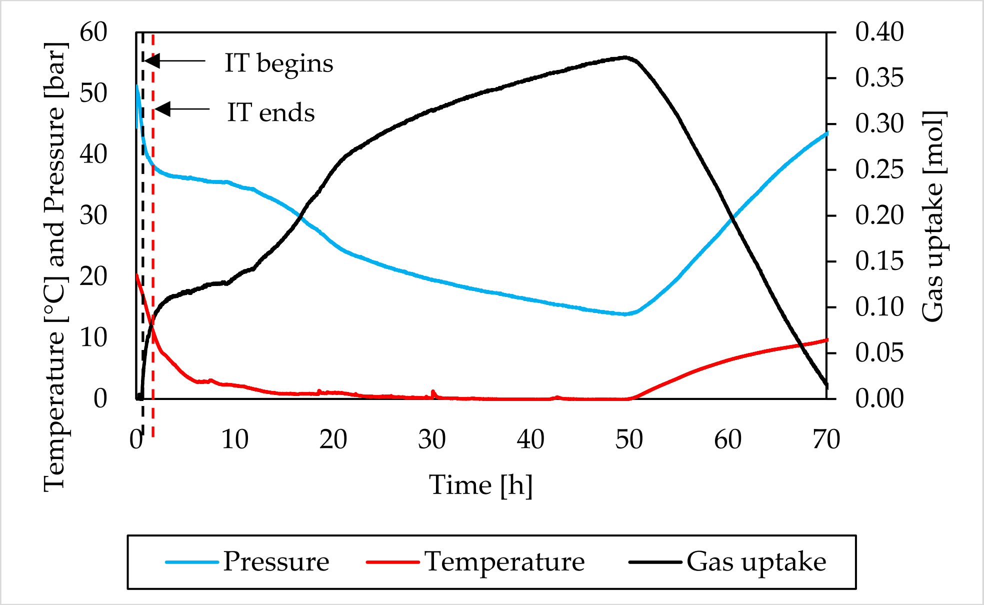

Figure S8. Pressure, temperature and gas consumption over time in Test G, carried out with carbon dioxide and in presence of 26.04 wt% Ti23 powder.
